# 17β-estradiol lowers triglycerides in adipocytes via estrogen receptor α and it may be attenuated by inflammation

**DOI:** 10.1186/s12944-017-0575-6

**Published:** 2017-09-25

**Authors:** Fei Luo, Wen-yu Huang, Yuan Guo, Gui-yun Ruan, Ran Peng, Xiang-ping Li

**Affiliations:** 1 Department of Cardiovascular Medicine, The Second Xiangya Hospital, Central South University, No.139 Renmin Middle Road, Changsha, 410011 Hunan People’s Republic of China; 2 Department of Emergency Medicine, Yantai Yuhuangding Hospital, Qingdao University Medical College, Yantai, Shangdong 264000 People’s Republic of China

**Keywords:** 17β-estradiol, Adipocyte, Triglyceride, Estrogen receptor α

## Abstract

**Background:**

Estrogen was reported to protect against obesity, however the mechanism remains unclear. We aimed to investigate the impact of 17β-estradiol (17β-E2) on triglyceride metabolism in adipocytes with or without lipopolysacchride (LPS) stimulating, providing novel potential mechanism for estrogen action.

**Methods:**

3T3-L1 adipocytes were cultured and differentiated into mature adipocytes in vitro. The differentiated 3T3-L1 cells were divided into six groups: (i) control group, treated with 0.1% DMSO alone; (ii) 17β-E2 group, treated with 1, 0.1, or 0.001 μM 17β-E2 for 48 h; (iii) 17β-E2 plus MPP group, pre-treated with 10 μM MPP (a selective ERα receptor inhibitor) for 1 h, then incubated with 1 μM 17β-E2 for 48 h; (iv) 17β-E2 plus PHTPP group, pre-treated with 10 μM PHTPP (a selective ERβ receptor inhibitor), then incubated with 1 μM 17β-E2 for 48 h; (v) LPS group, pre-treated with 100 ng/mL LPS for 24 h, then cells were washed by PBS for 3 times and incubated with 0.1% DMSO alone for 48 h; (vi) 17β-E2 plus LPS group, pre-treated with 100 ng/mL LPS for 24 h, then cells were washed by PBS for 3 times and incubated with 1 μM 17β-E2 for 48 h. The levels of triglyceride and adipose triglyceride lipase (ATGL) in differentiated 3T3-L1 cells and the concentrations of interleukin-6 (IL-6) in culture medium were measured.

**Results:**

Comparing with control group, 1 μM and 0.1 μM 17β-E2 decreased the intracellular TG levels by about 20% and 10% respectively (all *P* < 0.05). The triglyceride-lowing effect of 17β-E2 in differentiated 3T3-L1 cells was abolished by ERα antagonist MPP but not ERβ antagonist PHTPP. Comparing with control group, the IL-6 levels were significantly higher in the culture medium of the cultured differentiated 3T3-L1 cells in LPS group and 17β-E2 + LPS group (all *P* < 0.05). And, the IL-6 levels were similar in LPS group and 17β-E2 + LPS group (*P* > 0.05). There was no significant difference in the triglyceride contents of differentiated 3T3-L1 cells among control group, LPS group and 17β-E2 + LPS group (all *P* > 0.05). ATGL expression in 17β-E2 group was significantly higher than control group (*P* < 0.05), which was abolished by ERα antagonist MPP or LPS.

**Conclusions:**

17β-E2 increased ATGL expression and lowered triglycerides in adipocytes but not in LPS stimulated adipocytes via estrogen ERα.

## Background

Obesity is considered as an abnormal or excessive fat accumulation and it is one of the most important disturbances associated with the menopausal. The incidence of obesity in women at the age of 40 to 65 is up to 65% [1]. Previous studies reported the prevalence of obesity and metabolic syndrome in menopausal women is 3.3-fold higher among postmenopausal women than premenopausal women. Donato et al. reported postmenopausal women have five times the chance of having central adiposity than premenopausal women, even after controlling for BMI and other confounding factors [2]. And adiposity in postmenopausal women can be attenuated by estradiol replacement therapy [3]. In rodents, ovariectomy result in a significant weight gain and which can be reversed with the administration of exogenous estradiol [4–6]. Accordingly, this evidence indicates the rapid decline of estrogen levels contributes to obesity and estrogen can protect against obesity. However, the anti-obesity mechanism of estradiol is still unclear.

Obesity is ascribed to an imbalance between energy intake and expenditure and is characterized by increased storage of triglyceride (TG) in an expanded adipose tissue mass [7]. Inhibition of TG accumulation in adipocyte is an important key to attenuate obesity. Previous studies have reported estrogen could regulate plasma TG level [8]. Lack of estrogen in post-menopausal women leads to an increase in plasma TG and transdermal estradiol treatment for post-menopausal women could lower plasma TG levels by about 16.4% (*P* < 0.01) [9–11]. However, the anti-obesity effect of estradiol cannot be fully explained by its impact on plasma TG levels [6]. Besides, it was reported estradiol has apparent effect on intracellular TG metabolism beyond its intravascular effect. Study reported 17β-estradiol (17β-E2) reduces TG levels in the liver by inhibiting liver X receptor (LXR) activation [12]. Zhang et al. reported that estradiol significantly reduces TG accumulation in mouse embryonic fibroblasts [13]. This evidence indicated estradiol directly regulates intracellular TG accumulation, but the underlying mechanisms remain to be fully elucidated. It is believed that obesity is also a state of chronic inflammation [14]. Therefore, we wonder whether the inflammation will affect the effect of estradiol on TG in adipocyte. We designed the experiment to study the effects of estradiol on TG contents of adipocytes with or without inflammatory conditions in vitro.

## Methods

### Cell culture and differentiation

The 3T3-L1 cells were obtained from the American Type Culture Collection (ATCC, Rockville, USA) and cultured in Dulbecco modified eagle medium (DMEM, GIBICO, Invitrogen corporation, Changsha, china) supplemented with 10% fetal bovine serum (FBS, Sigma–Aldrich, St. Louis, MO, USA) at 37 °C under a humidified atmosphere of 5% CO2 and 95% air. Differentiation of 3T3-L1 cells to mature adipocytes was performed as previous described [15]. Briefly, 3T3-L1 cells were seeded at 6-well plate and stimulated with 0.4 mmol/L 3-isobutyl-1-methylxanthine (IBMX) (Sigma-Aldrich), 0.25 μmol/L dexamethasone (Dex, Sigma-Aldrich) and 10 μg/mL insulin (Sigma-Aldrich) in DMEM containing 10% FBS for 72 h. Then, cells were cultured with 10 μg/mL insulin alone in DMEM containing 10% FBS for another 8 d. The cell-culture medium was replenished every other day. Approximately 90% of the 3T3-L1 cells had differentiated into mature adipocytes on day 12.

### Cell treatment

Differentiated 3T3-L1 adipocytes were starved in serum free DMEM for 12 h and then were divided into six groups: (i) control group, treated with 0.1% DMSO alone; (ii) 17β-E2 group, treated with 1, 0.1, or 0.001 μM 17β-E2 (Abcam, Cambridge, UK) for 48 h; (iii) 17β-E2 plus MPP group, pre-treated with 10 μM MPP (a selective ERα receptor inhibitor, Tocris Bioscience, Bristol, UK) for 1 h, then incubated with 1 μM 17β-E2 for 48 h; (iv) 17β-E2 plus PHTPP group, pre-treated with 10 μM PHTPP (a selective ERβ receptor inhibitor, Tocris Bioscience, Bristol, UK), then incubated with 1 μM 17β-E2 for 48 h; (v) LPS group, pre-treated with 100 ng/mL lipopolysaccharide (LPS) for 24 h, then cells were washed by PBS for 3 times and incubated with 0.1% DMSO alone for 48 h; (vi) 17β-E2 plus LPS group, pre-treated with 100 ng/mL LPS for 24 h, then cells were washed by PBS for 3 times and incubated with 1 μM 17β-E2 for 48 h.

### Triglyceride and IL-6 quantification

Triglyceride contents in adipocytes and IL-6 levels in supernatant were separately measured using triglyceride assay kit (BioVision, Mannheim, San Francisco, USA) and IL-6 assay kit (Wuhan xinqidi Biological, Wu-Han, China) according to the manufacturers’ instructions.

### Western blotting

Western blotting was performed as previous described [16]. Briefly, the proteins from differentiated 3T3-L1 adipocytes were extracted with RIPA lysis buffer (Beyotime, Beijing, China) and separated on sodium dodecyl sulfate-polyacrylamide gel electrophoresis (SDS-PAGE) before transferring on to polyvinylidene difluoride (PVDF) membranes. Then, the membranes were blocked and then incubated with the following antibodies: mouse monoclonal anti-ATGL (Abcam, Cambridge) at 4°C for overnight. Blots were washed, incubated with secondary antibody, and visualized by chemiluminescence.

### Statistical analysis

All values are expressed as mean ± standard deviation (SD) and analyzed by One-way analysis of variance (ANOVA) and Student’s t-test, using SPSS 20.0 statistical software (SPSS Inc., Chicago, IL, USA). *P* < 0.05 (two sided) was considered as statistically significant.

## Results

### 17β-E2 lowers triglyceride content in differentiated 3T3-L1 cells

**Fig. 1:**
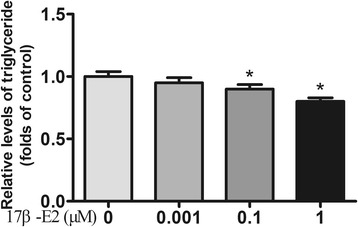
17β-E2 lowers triglyceride content in differentiated 3T3-L1 cells. Differentiated 3T3-L1 adipocytes were starved in serum free DMEM for 12 h and then were treated with 0.1% DMSO alone or different concentrations of 17β-E2 (1, 0.1, or 0.001 μM) for 48 h. Intracellular TG contents were normalized by total protein. The data were presented as the mean ± SD, *n* = 3 for each group. *P < 0.05, compared with Control group. TG: triglyceride

To investigate the effect of 17β-E2 on TG content of differentiated 3T3-L1 cells, the TG levels in adipocytes were determined after treatment with different concentrations of 17β-E2 for 48 h. We observed that 17β-E2 significantly decreased intracellular TG levels in a dose-dependent manner (Fig. 1).

### 17β-E2 lowers triglyceride content in differentiated 3T3-L1 cells possibly via ERα

**Fig. 2:**
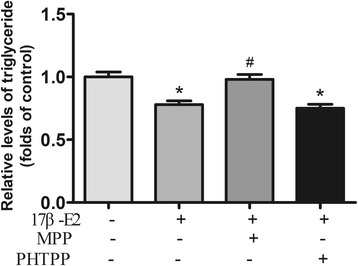
17β-E2 lowers triglyceride content in differentiated 3T3-L1 cells possibly via ERα. Differentiated 3T3-L1 adipocytes were starved in serum free DMEM for 12 h and then were treated with 0.1% DMSO alone, 1 μM 17β-E2, 10 μM MPP (pre-treated for 1 h) plus 1 μM 17β-E2 or 10 μM PHTPP (pre-treated for 1 h) plus 1 μM 17β-E2 for 48 h. Intracellular TG contents were normalized by total protein. The data were presented as the mean ± SD, *n* = 3 for each group. *P < 0.05, compared with Control group; #*P* < 0.05, compared with 17β -E2 group. MPP: a selective estrogen receptor α inhibitor, PHTPP: a selective estrogen receptor β inhibitor

Estrogen receptor α (ERα) and ERβ are the main estrogen receptors, we used selective receptor inhibitors to investigate the role of them in the TG-lowing effect of 17β-E2. The triglyceride-lowing effect of 17β-E2 in differentiated 3T3-L1 cells was abolished by ERα antagonist MPP, while ERβ antagonist PHTPP did not significantly affect the TG-lowing effect (Fig. 2).

### 17β-E2 did not affect triglyceride content in differentiated 3T3-L1 cells on inflammatory state

**Fig. 3:**
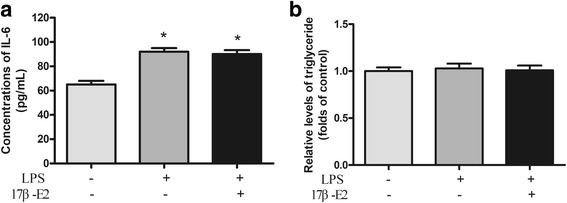
17β-E2 did not affect triglyceride content in differentiated 3T3-L1 cells on inflammatory state. Differentiated 3T3-L1 adipocytes were starved in serum free DMEM for 12 h and then were treated with 0.1% DMSO alone, 100 ng/mL LPS (pre-treated for 24 h then washed with PBS), 100 ng/mL LPS (pre-treated for 24 h then washed with PBS) plus 1 μM 17β-E2 for 48 h. Intracellular TG contents were normalized by total protein. **a**) Concentrations of IL-6; **b**) Relative levels of triglyceride. The data were presented as the mean ± SD, *n* = 3 for each group. *P < 0.05, compared with Control group. LPS: lipopolysaccharide

Inflammation and its failure to resolve are firmly established as central to the development and complications of several cardiovascular diseases [17]. We used the LPS-stimulated 3T3-L1 cell line as an inflammatory model and evaluated the effect of 17β-E2 on TG content of differentiated 3T3-L1 cells when exposed to LPS. LPS to induce inflammation response, as shown in Fig. 2, LPS (100 ng/mL) significantly increased IL-6 levels in feeder cell culture supernatants in LPS group and 17β-E2 + LPS group, compared with control group (Fig. 3a). There was no significant difference between control group, LPS group and LPS + 17β-E2 group for TG content of differentiated 3T3-L1 cells (Fig. 3b).

### 17β-E2 increased ATGL expression in differentiated 3T3-L1 cells via ERα

**Fig. 4:**
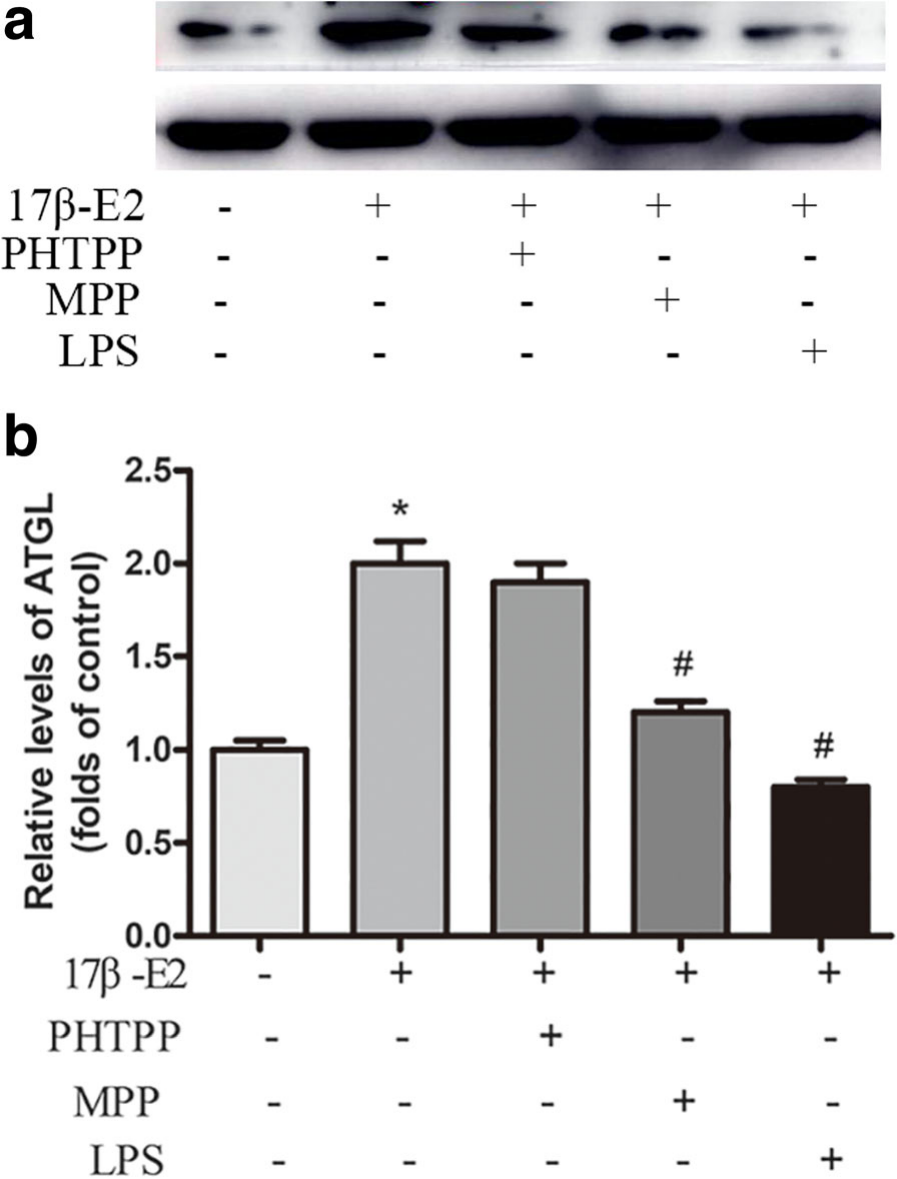
17β-E2 increased ATGL expression in differentiated 3 T3-L1 cells via ERα. **a**) Expression of ATGL was analyzed by western blot. **b**) Relative expression of ATGL. The data were presented as the mean ± SD, *n* = 3 for each group. *P < 0.05, compared with Control group; #*P* < 0.05, compared with 17β -E2 group. MPP: a selective estrogen receptor α inhibitor, PHTPP: a selective estrogen receptor β inhibitor

Adipose triglyceride lipase (ATGL) is an important regulator in TG hydrolysis. We evaluated the effect of 17β-E2 on ATGL expression. We found ATGL expression in 17β-E2 group was significantly higher than control group (Fig. 4). The upregulation of ATGL induced by 17β-E2 was abolished by ERα antagonist MPP or LPS (Fig. 4).

## Discussion

The accumulation of TG in adipocytes is associated with metabolic diseases including obesity. Ding et al. reported ovariectomy significantly increases adipose tissue mass [6]. Deficiency of aromatase, the enzyme responsible for estrogen biosynthesis, results in increased adiposity [18], indicating estrogen could regulate adipocyte. Zhang et al. [13] reported estradiol inhibits the adipocyte marker genes and adipogenesis. Our present study showed that 17β-E2 significantly decreased the TG content in differenced 3T3-L1 cells.

ERα and ERβ are the main classical receptors of estrogen and are reported to express in 3T3-L1 cells. Previous studies reported that ERα plays important role in lipids and glucose metabolism [19, 20]. Wend et al. [21] found bone marrow cells from ERα knockout (KO) mice differentiated to adipocytes in vitro have increased lipid accumulation compared to cells from wild-type mice or ERβ KO mice. Pedram et al. [22] found 17β-E2 bound membrane ERα and then repressed TG contents in differentiated adipocytes. These evidences indicated ERα but not ERβ plays a pivotal role in estradiol induced TG-lowering effect. Our present result, for the first time, found 17β-E2 decreased TG through ERα but not ERβ.

ATGL is a key enzyme involved in intracellular degradation of TG. ATGL catalyzes the first step of TG catabolism which hydrolyzes TG in diacylglycerol [23]. Wohlers et al. [24] found expression of ATGL in visceral fat is not affected with estrogen treatment in ovariectomized mice. But, the results of animal experiment may be affected by multiple factors such as the dose and duration of estrogen treatment. We found 17β-E2 significantly increased expression of ATGL in vitro. Recent study reported bone marrow cells from ERα KO mice differentiated to adipocyte in culture have lower expression of ATGL than wild-type adipocyte and estradiol treatment cannot affect the expression of ATGL [21]. It indicated that estradiol via ERα upregulates ATGL expression. Therefore, 17β-E2 may decrease TG accumulation through increasing the expression of ATGL.

Obesity is an important risk factor of atherosclerosis [25, 26]. Early estradiol replacement therapy in postmenopausal women was demonstrated to be efficient to attenuate the progress of atherosclerosis [27]. However, the Heart and Estrogen/progestin Replacement Study (HERS) and the Estrogen Replacement and Atherosclerosis (ERA) study found estrogen replacement treatment did not reduce risk of cardiovascular events in women with coronary artery atherosclerosis (CAD) [28–30]. Atherosclerosis is a chronic disease of the arterial wall largely driven by inflammation [31]. Previous studies indicated estrogen did not attenuate the progress of atherosclerosis in women who had already developed atherosclerosis [32, 33]. Therefore, we speculate that inflammation may affect the effect of estradiol. In present study, we further evaluated the effect of estradiol on TG contents on inflammatory state induced by LPS. We found that IL-6 levels in feeder cell culture supernatants were significantly increased when 3T3-L1 exposed to LPS, it indicated LPS could induce inflammation. For the first time, we found 17-E2 did not decrease TG contents or increase expression of ATGL in LPS-stimulated adipocytes. The result indicated that the TG-lowering effect in adipocytes is abolished by inflammation. The underlying mechanisms deserve further investigation.

## Conclusion

This study illustrated 17β-E2 can increase ATGL expression and lower triglycerides in adipose cells via ERα and this effect of 17β-E2 was attenuated by LPS stimulation. It suggests that the TG-regulating effect of 17β-E2 on adipocytes may be affected by inflammation. Further studies are needed to explore the underlying mechanisms.

## Abbreviations

17β-E2: 17β-estradiol
ATGL: Adipose triglyceride lipase
CAD: Coronary artery disease
DMEM: Dulbecco modified eagle medium
ERA: Estrogen Replacement and Atherosclerosis
ERα: Estrogen receptor α
ERβ: Estrogen receptor β
FBS: Fetal bovine serum
HERS: Heart and Estrogen/progestin Replacement Study
KO: Knockout
LPS: Lipopolysaccharide
TG: Triglyceride
